# Cannabis use frequency, route of administration, and co-use with alcohol among older adults in Washington state

**DOI:** 10.1186/s42238-021-00071-3

**Published:** 2021-06-03

**Authors:** Meenakshi S. Subbaraman, William C. Kerr

**Affiliations:** Alcohol Research Group, Public Health Institute, 6001 Shellmound Ave, Suite 450, Emeryville, CA 94608 USA

**Keywords:** Cannabis, Marijuana, Frequency, Route of administration, Alcohol, Older adults

## Abstract

**Background:**

The US national surveys and data from legal adult use cannabis states show increases in the prevalence of cannabis use among older adults, though little is known about their manner of cannabis consumption. Here, we examine cannabis use frequency, routes of cannabis administration, and co-use with alcohol, focusing on adults aged 50–64 and ≥65.

**Methods:**

Data come from a general population survey conducted January 2014–October 2016 (N=5492) in Washington state. We first estimate prevalence and trends in cannabis frequency, routes of administration, and co-use with alcohol in gender by age groups (18–29, 30–49, 50–64, ≥ 65). To test associations between cannabis frequency, route of administration, and co-use with alcohol, we then use sample-weighted multinomial regression adjusted for gender, race/ethnicity, marital status, education, employment, and survey year. Sampling weights are used so results better represent the Washington state population. Regressions focus on the 50–64 and ≥65 age groups.

**Results:**

Among men and women 50–64, the prevalence of no cannabis use in the past 12 months decreased significantly (84.2% in 2014 to 75.1% in 2016 for women, 76.8% in 2014 to 62.4% in 2016 for men). Among those who report past-year cannabis use, oral administration and vaping and other routes of administration increased by 70% and 94%, respectively each year. Almost one-third of women aged 50–64 and one-fifth of women aged ≥65 who use cannabis reported daily/near daily use, and more than one-third of men who use cannabis in all age groups reported daily/near daily use, including 41.9% of those ≥65. Among men, the prevalence of edibles, drinks, and other oral forms of cannabis administration went up significantly with age (6.6% among 18–29, 21.5% among ≥65). Vaping and other administration are more strongly related to regular and daily/near daily use than infrequent use among those ≥65. The pattern of associations between cannabis frequency and co-use with alcohol differed for women vs. men.

**Conclusions:**

In a general population representative sample of adults living in a state with legal adult use cannabis, the prevalence of cannabis use increased among those aged 50–64 between 2014 and 2016, the prevalence of daily use is substantial, and oral administration and vaping are increasing.

## Key points

- Among adults 50-64 who use cannabis, 34.4% of women and 39.2% of men report daily/near daily use
- Among adults ≥65 who use cannabis, 17.9% of women and 41.9% of men report daily/near daily use
- Oral administration and vaping are increasing among adults 50-64
- Regular cannabis use is related to co-use with alcohol among men ≥65

## Introduction

Estimates from the National Survey on Drug Use and Health (NSDUH) indicate that the prevalence of past-year cannabis use among adults age ≥65 in the USA increased seven-fold over 10 years, jumping from 0.4% in 2006–2007 to 2.9% in 2015–2016 (Han and Palamar 2018). More recent NSDUH surveys similarly find that from 2015 to 2018, the prevalence of past-year cannabis use among adults age ≥65 rose from 2.4 to 4.2% (Han and Palamar 2020). Data from older adults in states with legalized adult use cannabis are limited, though analyses of surveys from Washington state, which did not separate adults ≥65 from those ≥50, also show significant increases in the past 12-month cannabis use among adults ≥50, which rose from 15.1% in 2014 to 23.6% in 2016, as well as simultaneous use of cannabis and alcohol, which rose from 6.1% in 2014 to 10.7% in 2016 (Subbaraman and Kerr 2020).

Detailed measures such as cannabis use quantity and frequency are primary indicators of cannabis-related and other health problems (Walden and Earleywine 2008), which makes understanding patterns of cannabis use beyond dichotomous “any use” measures of use prevalence crucial for preventing problems among older adults. However, few studies have examined cannabis use measures beyond the prevalence of any use in this population. A NSDUH study of 5325 adults aged ≥50 found that 23% of past-year users had used cannabis on at least half the days of the year (DiNitto and Choi 2011). A recent review (Lloyd and Striley 2018) of cannabis use among older adults cited several studies linking any past-year cannabis use and past-year use of other substances, including alcohol, tobacco, and other illicit drugs, and misuse of prescription drugs (DiNitto and Choi 2011; Choi et al. 2016; Han et al. 2017; Salas-Wright et al. 2017). However, the one study of cannabis use frequency and other drug use showed similar cannabis use frequency between those who used cannabis only and those who used cannabis with other drugs (Choi et al. 2016). Cannabis use frequency and alcohol co-use were not examined.

Relevant to co-use, NSDUH results show increased cannabis use among adults ≥65 who also use alcohol (Han et al. 2017) and significantly higher odds of alcohol use disorders among older individuals who use cannabis vs. those who do not (Han and Palamar 2018). Given the elevated risks associated with co-use vs. using either substance alone, NSDUH investigators encourage future research to monitor and educate older adults regarding co-use of cannabis and other substances (Han and Palamar 2020). Co-use of alcohol and cannabis is related to increased risk of alcohol-related harms (e.g., financial, health), social consequences (e.g., fights, work problems, legal issues), and risk behaviors, such as drunk-driving (Yurasek et al. 2017; Subbaraman and Kerr 2015). While these risks have been documented in the general population (Yurasek et al. 2017; Subbaraman and Kerr 2015) and among adolescents and young adults (Terry-McElrath et al. 2013; D'Amico et al. 2016; Patrick et al. 2018), the prevalence of co-use and how it relates to cannabis frequency among older adults is currently unknown. Older adults have more chronic diseases than younger adults and may be more susceptible to injury or other negative consequences while under the influence due to the physiological effects of aging (Lloyd and Striley 2018; Dowling et al. 2008), combined with the stronger potency of cannabis currently on the market (Han and Palamar 2018). Thus, understanding the prevalence of cannabis and alcohol co-use and how it relates to cannabis use frequency among older adults can help inform public health and clinical efforts.

Understanding common routes of cannabis administration among older adults can also help prevent adverse effects, e.g., through patient education regarding proper dosing. One review of cannabis administration and dosing notes that most significant interactions between cannabis and other drugs are attributable to concurrent use of other central nervous system depressants, like alcohol, and that onset and duration of effects vary widely by route of administration, with oral methods specifically having dosing titration challenges due to delayed onset of effects (MacCallum and Russo 2018). Another review of cannabis-prescription drug interactions recommends closely monitoring older individuals who use cannabis and those with chronic diseases or kidney and liver conditions (Alsherbiny and Li 2019). This is especially important as more states and jurisdictions continue to legalize medical and adult cannabis use, and the availability of cannabis-related products continues to diversify. However, data on cannabis routes of administration among older adults in legalized adult cannabis use states have only recently become available.

Washington was one of the first states to legalize adult cannabis use in 2012 and is therefore one of the few states with sufficient data for examining trends in cannabis use in the post-legalization period. Thus, the aims of this study are to describe and compare trends in the frequency of cannabis use, routes of administration, and co-use with alcohol among individuals aged 50–64 and ≥65.

## Methods

### Data

Data come from the *Effects of Spirits Privatization on Alcohol Prices and Alcohol-Related Harms* study (Subbaraman and Kerr 2020; Greenfield et al. 2018; Ye and Kerr 2016; Williams et al. 2020; Subbaraman et al. 2020; Subbaraman and Kerr 2016), a general population survey of Washington residents aged 18+. The overarching aim of the parent study was to examine the impacts of Washington’s policy change from government-controlled to privatized spirits retail sales on alcohol purchasing, consumption, and related problems in the general population. The primary variables collected were demographics, alcohol purchasing, alcohol use patterns, and alcohol policy-related opinions. Secondary variables included cannabis use. Data were collected in six separate cross-sectional samples across six time-points (every 6 months) between January 2014 and October 2016. Participants (*N* = 5492) were recruited via list-assisted dual-frame random digit dial procedures, with > 40% from cell phones. Cooperation rates were 50.8% (landline) and 59.5% (cell phone) for T1 (*N*=1202); 45.8% (landline) and 62.4% (cell) for T2 (*N*=804); 43.7% (landline) and 61.5% (cell) for T3 (*N*=823); 41.7% (landline) and 59.6% (cell) for T4 (*N*=662); 49.4% (landline) and 60.9% (cell) for T5 (*N*=610); and 45.3% (landline) and 63.0% (cell) for T6 (*N*=1391); the American Association for Public Opinion Research has detailed formulas for cooperation rates on their website (The American Association for Public Opinion Research 2000). Those who did not answer questions on cannabis use were excluded (1% of total sample). The Public Health Institute’s Institutional Review Board approved this study and waived written consent, and all participants gave oral informed consent.

*Frequency of cannabis use* was determined using the question, “How often have you used marijuana, hash or pot during the last twelve months?” Was it… (1) every day or nearly every day, (2) about once a week, (3) once every 2 or 3 weeks, (4) once every month or two, (5) less often than that, or (6) never. To improve statistical power and interpretability of results, cannabis use frequency was categorized as (1) infrequent use (less often than monthly), (2) regular use (once every month or two, once every 2 or 3 weeks, about once a week), and (3) daily/near daily use (every day or nearly every day).

Among individuals who use cannabis (n=1200), *most common route of administration* was determined from the question, “How do you most commonly consume marijuana?” Possible response categories included (1) smoke marijuana only, (2) smoke marijuana with tobacco, (3) vaporize marijuana, (4) smoke hashish or resin alone, (5) smoke hashish or resin with marijuana plant, (6) oil, wax, or dabs through inhalation, (7) eat food product (ex. brownies or candy), (8) drink tea or other beverage infused with cannabis, (9) use a cannabis tincture consumed orally, (10) apply lotion, salve, balm or spray, and (11) other. Route of administration was re-categorized for analyses as (1) smoke marijuana only, (2) vaporize marijuana, (3) oral ingestion (eat food, drink, oral tincture), and (4) other (remaining categories). *Co-use of cannabis and alcohol* was determined from the question, “In the past year, how often did you use alcohol and marijuana or marijuana products at the same time? Was it usually, sometimes, or never?” As done in our previous studies (Subbaraman and Kerr 2020; Subbaraman and Kerr 2015), those who answered “never” were grouped and those who answered “usually” or “sometimes” were grouped. The reference category was cannabis use/no alcohol use, making three cannabis/alcohol co-use groups.

### Statistical analyses

We first describe the prevalence of and trends in cannabis use frequencies, routes of administration, and co-use with alcohol across groups defined by age (18–29, 30–49, 50–64, ≥ 65) and gender. We then use multinomial regression where the primary exposure is cannabis use frequency and primary outcomes are route of administration and co-use of cannabis with alcohol. Multinomial regression models are further adjusted for race/ethnicity, marital status, education, and employment as covariates. Analyses are stratified by gender where sample sizes allow because men and women have different patterns of cannabis use and problems (Calakos et al. 2017; Substance Abuse and Mental Health Services Administration, Center for Behavioral Health Statistics and Quality 2014). Preliminary analyses confirmed significant interactions between gender and age in predicting cannabis use frequency in this sample, further justifying stratification by gender. All bivariate and multivariable analyses account for probability of selection due to the sampling design through survey weights. Sampling weights account for differential probability of response between landline and cell phone samples, and incorporate post-stratification weights for age, gender, race/ethnicity, and educational attainment based on the Washington 2010 Census. Sampling weights are used so results better represent the Washington state population. All analyses were performed in Stata V.16.1, StataCorp, College Station, TX, USA.

## Results

### Past 12-month prevalence in frequency, route of administration, and co-use with alcohol across age groups

In the overall sample, 55.8% of respondents were women and 84.7% were white. Among those who used cannabis in the past 12 months, 48.5% were women and 81.9% were white. Table 1 describes prevalence of cannabis frequencies, most common routes of administration, and co-use with alcohol across groups defined by gender and age. Older age groups had significantly (*P* < 0.001) lower prevalence of any cannabis than younger age groups among both women and men; the overall proportion of adults ≥65 who reported any use was relatively small (~9% of women and ~16% of men). There were no significant differences in cannabis use frequency or most common route of consumption across age groups among women. More than a third of men who use cannabis in all age groups reported daily/near daily use, i.e., 38.6 (18–29) to 49.1% (30–49), with significant differences across ages. Among both men and women, the prevalence of edibles, drinks, and other oral forms of cannabis administration went up with age though differences were significant across ages only for men. There were no significant (*P* < 0.05) differences in prevalence of co-use with alcohol across age groups.

**Table 1:** Past 12-month prevalence of cannabis use frequency, route of administration, and co-use with alcohol across gender by age subgroups, 2014–2016 Washington state general population (*N* = 5429)

	Women				*P* for difference across ages
	18–29	30–49	50–64	≥65	
	(*n*=330)	(*n*=753)	(*n*=947)	(*n*=1037)	
Never used cannabis in past the 12 months (%)	**62.2**	**75.1**	**76.5**	**91.5**	**< 0.001**
n of past 12-month cannabis users	111	174	200	97	
Frequency among past 12-month cannabis users (%, *n*=582)					
Infrequent^a^	28.0	32.7	27.0	46.8	0.36
Regular	38.9	33.5	38.6	35.3	
Daily/near daily	33.0	33.8	34.4	17.9	
Most frequent route of administration (%)					
Smoke only	73.9	73.2	64.9	58.7	0.54
Vaporize	4.9	8.6	8.9	11.2	
Edibles, drinks, oral	15.6	14.1	17.6	25.9	
Other	5.7	4.0	8.5	4.1	
Co-use with alcohol (%)					
Cannabis only/no alcohol	9.2	14.3	24.5	13.3	0.06
Always uses separately	42.2	41.8	36.2	51.6	
Usually/always	48.6	43.9	39.3	35.1	
	Men				*P* for difference
	18–29	30–49	50–64	≥65	
	(*n*=349)	(*n*=624)	(*n*=747)	(*n*=705)	across ages
Never used cannabis in past the 12 months (%)	**52.3**	**67.3**	**70.3**	**83.8**	**< 0.001**
n of past 12-month cannabis users	153	176	196	93	
Frequency among past 12-month cannabis users (%, *n*=618)					
Infrequent	19.8	24.3	20.7	23.2	0.23
Regular	41.5	26.7	40.2	34.9	
Daily/near daily	38.6	49.1	39.2	41.9	
Most frequent route of administration (%)					
Smoke only	**79.4**	**74.6**	**79.1**	**56.6**	**0.008**
Vaporize	**10.4**	**11.9**	**6.2**	**12.9**	
Edibles, drinks, oral	**6.6**	**5.9**	**12.6**	**21.5**	
Other	**3.7**	**7.6**	**2.1**	**9.0**	
Co-use with alcohol (%)					
Cannabis only/no alcohol	9.2	16.2	22.5	25.7	0.13
Always uses separately	32.6	27.8	26.7	32.8	
Usually/always	58.2	56.0	50.8	41.5	

^a^Infrequent =< monthly; regular = monthly, > monthly/< weekly, weekly; daily/near daily = daily

Bold indicates *P* for difference across age groups < 0.05

### Trends in frequency, route of administration, and co-use with alcohol

Table 2 shows time trends in frequencies, routes of administration, and co-use with alcohol across groups defined by gender and age. Among women 50–64, the prevalence of no cannabis use in the past 12 months decreased significantly (*P* < 0.01) from 84.2% in 2014 to 75.1% in 2016. Among men 50–64, the prevalence of no cannabis use in the past 12 months decreased significantly (*P* < 0.05) from 76.8% in 2014 to 62.4% in 2016. There were no significant trends in cannabis use frequency of route administration for women or men, though the prevalence of regular cannabis use among women rose from 31.8 to 50.3% (*P* < 0.08).

**Table 2:** Trends in prevalence (%) of past 12-month cannabis use across gender by age groups, 2014–2016 Washington state general population (*N* = 5429)

	WOMEN								MEN							
	50–64				≥65				50–64				≥65			
	2014	2015	2016	*P*	2014	2015	2016	*P*	2014	2015	2016	*P*	2014	2015	2016	*P*
Never used cannabis in the past 12 months (%)	**84.2**	**71.3**	**75.1**	**0.01**	93.1	93.4	88.2	0.07	**76.8**	**71.9**	**62.4**	**0.04**	85.3	86.7	81.8	0.59
Frequency among past 12-month cannabis users (%)																
Infrequent^a^	18.2	35.7	23.2	0.08	51.9	47.9	43.3	0.98	25.5	15.8	21.6	0.76	32.8	2.3	30.6	0.20
Regular	31.8	31.7	50.3		30.6	35.1	38.1		34.1	47.4	38.0		26.7	48.8	31.4	
Daily/near daily	50.0	32.6	26.5		17.5	17.0	18.6		40.4	36.8	40.3		40.5	48.9	38.0	
Most common route of administration (%)																
Smoke only	78.8	61.8	58.7	0.52	67.6	59.5	53.8	0.52	92.9	75.7	74.5	0.31	56.2	43.2	66.7	0.86
Vaporize	7.9	9.6	8.8		3.6	6.2	18.0		3.4	6.8	7.3		10.3	16.8	12.0	
Oral	11.3	19.0	20.5		28.8	27.9	23.3		3.7	16.0	14.7		24.0	27.6	15.2	
Other	2.0	9.5	12.0		0.0	6.4	4.9		0.0	1.6	3.5		9.5	12.4	6.1	
Co-use with alcohol (%)																
Cannabis only/no alcohol	**22.8**	**13.7**	**36.9**	**0.01**	12.2	19.2	10.5	0.42	24.9	20.8	22.5	0.50	32.0	26.4	20.5	0.50
Always uses separately	**27.9**	**53.2**	**23.7**		66.5	36.3	52.6		36.7	20.2	26.0		30.0	46.4	25.5	
Usually/always	**49.3**	**33.2**	**39.4**		21.3	44.5	36.9		38.5	59.0	51.4		38.1	27.2	54.0	

^a^Infrequent = < monthly; regular = monthly, > monthly/< weekly, weekly; daily/near daily = daily

Bold indicates *P* for trend < 0.05

### Relationship between cannabis use frequency and route of administration

Table 3 displays relative risk ratios (RRR) from age-stratified multinomial regression models of past 12-month cannabis use frequency regressed on route of cannabis administration (where smoking only is the referent). Genders were combined for this analysis due to sample size constraints, so regression models adjust for gender as a covariate instead. Among those aged 50–64, the relative risk of preferring oral administration vs. smoking only is 0.21 times lower for daily/near daily use vs. infrequent use (*P* < 0.003), and the risk of oral administration vs. smoking only increases 70% each year (*P* < 0.03). The risk of vaping and other routes of administration vs. smoking only increases 94% each year among those age 50–64 (*P* < 0.007). Among those ≥65, the relative risk of preferring vaping and other administration vs. smoking only is 5.36 times higher for regular use vs. infrequent use (*P* < 0.015) and 6.77 times higher for daily/near daily use vs. infrequent use (*P* < 0.007).

**Table 3:** Results from multinomial regression models^a^ of past 12-month cannabis use frequency regressed on route of cannabis administration, 2014–2016 Washington state general population (*N* = 5429)

	Overall															
	50–64								≥65							
	Oral^b^				Vape and other^b^				Oral				Vape and other			
	RRR	95% CI		*P*	RRR	95% CI		*P*	RRR	95% CI		*P*	RRR	95% CI		*P*
Frequency among past 12-month cannabis users (ref = infrequent^c^)																
Regular	1.01	0.43	2.35	0.98	1.36	0.48	3.85	0.56	0.77	0.28	2.16	0.63	**5.36**	**1.38**	**20.76**	**0.015**
Daily/near daily	**0.21**	**0.07**	**0.58**	**0.003**	0.83	0.29	2.37	0.73	0.49	0.15	1.65	0.25	**6.77**	**1.68**	**27.30**	**0.007**
Year	**1.70**	**1.04**	**2.77**	**0.03**	**1.94**	**1.20**	**3.13**	**0.007**	0.72	0.42	1.23	0.23	1.27	0.70	2.31	0.43

^a^Regressions adjust for gender, race/ethnicity, marital status, education, and employment. Relative risk ratios (RRR) are interpreted, e.g., among those 50–64, the relative risk of preferring oral administration vs. smoking only is 0.21 times lower for daily/near daily use vs. infrequent use (*P* < 0.003)

^b^Ref = smoke cannabis only

^c^Infrequent = < monthly; regular = monthly, > monthly/< weekly, weekly; daily/near daily = daily

Bold indicates *P* < 0.05

### Relationship between cannabis use frequency and co-use with alcohol

Table 4 displays results from multinomial regression models of past 12-month cannabis frequency regressed on co-use of cannabis and alcohol across groups defined by gender and age. Among women 50–64, the relative risk of always using cannabis and alcohol separately vs. using cannabis only (no alcohol) is 0.20 times lower for regular vs. infrequent use (*P* < 0.01) while the risk of sometimes/usually using cannabis and alcohol together was 7.09 times higher for daily/near daily vs. infrequent use (*P* < 0.01). The pattern of results differed for men: among men ≥65, the risk of sometimes/usually using cannabis and alcohol together was 9.51 times higher for regular vs. infrequent use (*P* < 0.01). There were no significant changes in co-use over time for women or men in multivariable models.

**Table 4:** Results from multinomial regression models^a^ of past 12-month cannabis use frequency regressed on co-use of cannabis and alcohol, 2014–2016 Washington state general population (*N* = 5429)

	Women															
	50–64								≥65							
	Always uses separately^b^				Sometimes/usually uses together^b^				Always uses separately				Sometimes/usually uses together			
	RRR	95% CI		*P*	RRR	95% CI		*P*	RRR	95% CI		*P*	RRR	95% CI		*P*
Frequency among past 12-month cannabis users (ref = infrequent^c^)																
Regular	**0.20**	**0.06**	**0.67**	**0.01**	2.40	0.69	8.41	0.17	2.15	0.43	10.71	0.35	3.20	0.55	18.52	0.19
Daily/near daily	0.82	0.18	3.68	0.80	**7.09**	**1.58**	**31.82**	**0.01**	1.54	0.22	10.79	0.66	4.13	0.54	31.72	0.17
Year	0.68	0.34	1.38	0.29	0.73	0.35	1.54	0.41	0.75	0.30	1.86	0.53	1.11	0.44	2.75	0.83
	Men															
	50–64								≥65							
	Always uses separately^b^				Sometimes/usually uses together				Always uses separately				Sometimes/usually uses together			
	RRR	95% CI		*P*	RRR	95% CI		*P*	RRR	95% CI		*P*	RRR	95% CI		*P*
Frequency among past 12-month cannabis users (ref = infrequent^c^)																
Regular	0.45	0.12	1.67	0.23	1.74	0.46	6.51	0.41	1.91	0.39	9.23	0.42	**9.51**	**1.33**	**68.15**	**0.03**
Daily/near daily	0.28	0.07	1.17	0.80	1.28	0.34	4.78	0.72	0.88	0.12	6.47	0.90	3.04	0.36	25.72	0.31
Year	0.88	0.44	1.78	0.72	1.10	0.58	2.09	0.77	1.04	0.51	2.14	0.92	1.65	0.63	4.30	0.30

^a^Regressions adjust for gender, race/ethnicity, marital status, education, and employment. Relative risk ratios (RRR) are interpreted as, e.g., among women 50–64, the relative risk of always using cannabis and alcohol separately vs. using cannabis only (no alcohol) is 0.20 times lower for the regular vs. infrequent users (*P* < 0.01)

^b^Ref = cannabis only/no alcohol

^c^Infrequent = < monthly; regular = monthly, > monthly/< weekly, weekly; daily/near daily = daily

Bold indicates *P* < 0.05

### Post hoc corrections for multiple testing

Post hoc analyses examined *P*-values using the Benjamini-Hochberg approach (Benjamini and Hochberg 1995) with false discovery rates (FDR) of 0.25 and 0.05 and Bonferroni corrections (α= 0.05/60=0.00083) to account for multiple comparisons. Under Benjamini-Hochberg with FDR = 0.25, all significant results reported above remained significant. When using Benjamini-Hochberg with FDR = 0.05 or Bonferroni corrections, only the differences in never having used cannabis across age groups remained statistically significant.

## Discussion

We find that in 2014–2016, the years following adult use cannabis legalization in Washington State, cannabis use differs across age groups, with high levels of daily/near daily (daily) use reported among individuals aged 50–64 and ≥65. Though older age groups had lower prevalence of any cannabis use than younger groups, the prevalence of cannabis use increased among both women and men aged 50–64 specifically during the study time period. Published results from Washington show that 12-month cannabis use among adults ≥50 increased from the same 2014–2016 period (Subbaraman and Kerr 2020), and current results clarify that this increase was primarily driven by those 50–64 years old with no significant changes among those ≥65. The 50–64-year-old age group consists entirely of baby boomers, so the increase in cannabis use over time is likely a period effect facilitated by higher past use rates among this cohort. Age-period-cohort analyses of the 2002–2015 NSDUH show that compared to the 1940s birth cohort, the 1950s birth cohort had 1.8 times the prevalence of past-month cannabis use (*P* < 0.05), though cohort-specific trends were not reported (Calakos et al. 2017). Similarly, age-period-cohort analyses of the National Alcohol Survey found that the increase in the national prevalence of cannabis use among older people between 1984 and 2015 can be attributed to both cohort and period effects (Kerr et al. 2018).

Almost one-third of women aged 50–64 and one-fifth of women aged ≥65 who use cannabis reported daily/near daily use, and more than one-third of men who use cannabis in all age groups reported daily/near daily use, including 41.9% of those ≥65. The prevalence of daily/near daily use is thus becoming more similar between genders in the 50–64 baby boomer cohort, which is not surprising due to their higher rates of substance use and different attitudes towards cannabis compared to earlier generations (Han et al. 2017; Blazer and Wu 2009). The 2002–2014 NSDUH similarly showed that among adults 50–64, the prevalence of daily use tripled from 2007 to 2014 and increases in nondaily cannabis use were larger than all other age groups from 2002 to 2014 (Mauro et al. 2018), though gender was not examined.

The use of edibles, drinks, and other oral forms of cannabis administration was most prevalent among older ages, and though not significant in bivariate trend models, both oral administration and vaping increased significantly over time among those aged 50–64 in multivariable models. The increase in oral forms might be of concern as a recent study of treatment protocols for medical cannabis noted that oral preparations are not recommended for older adults because of the delayed onset in effects and difficulties in achieving stable effects (Abuhasira et al. 2019). Focus groups in Washington and Colorado similarly report that the effects of edibles and other forms of orally ingestible cannabis can be unpredictable and inconsistent (Giombi et al. 2018), and that advice regarding consumption is often unclear from the packaging (Kosa et al. 2017). For example, a qualitative study of medical and recreational cannabis use by older adults in Colorado found lack of education and research about cannabis and lack of provider communication as common themes, and concluded that adults want more information and communication with their healthcare providers about cannabis (The American Association for Public Opinion Research 2000). Thus, there may be a need for patient education around dosing oral cannabis among older adults, especially relative to potential alcohol and prescription drug interactions.

Among women 50–64, the use of cannabis/not using any alcohol increased significantly over time while simultaneous use of cannabis and alcohol did not change. Among women ≥65 and men 50–64 and ≥65, simultaneous use did increase, though trends were not statistically significant. Still, current results support earlier results showing increases in simultaneous use among adults ≥50 in Washington (Subbaraman and Kerr 2020) and extend earlier results by further disaggregating by gender. Current results also show that co-use is significantly related to cannabis use frequency. Among women aged 50–64, regular use was related using cannabis only (no alcohol), and daily/near daily use was related to sometimes/usually using cannabis and alcohol together. The differential associations between cannabis frequency and co-use with alcohol may reflect different motivations for use, e.g., heavier cannabis combined with alcohol co-use could signal intoxication as a motivation for use. However, this survey did not collect data regarding motivations, so future studies should consider including questions regarding motivations for substance use among older adults. Among men ≥65, regular cannabis use was related to highest risk of sometimes/usually using cannabis and alcohol together. The high prevalence of regular use among men (34.9%) combined with the strong relationship between regular use and using cannabis and alcohol together is particularly concerning as simultaneous use is linked to increased likelihood of alcohol-related problems compared to using alcohol alone (Yurasek et al. 2017; Subbaraman and Kerr 2015; Subbaraman and Kerr 2018). Ongoing studies are examining whether, for example, co-use of cannabis and alcohol cluster with other risky behaviors as correlates of health conditions among older adults.

Individuals ≥50 in NSDUH who had used cannabis in the past year had significantly higher psychological distress scores, though they did not rate their health as worse than others in the sample, nor did they attribute many problems, including psychological problems, as related to cannabis use (DiNitto and Choi 2011). Here, we found that individuals ≥50 who use cannabis may be at particularly high risk of health problems because, in addition to frequent cannabis use, they also are more likely to engage in other risky substance use like co-use with alcohol. Significantly more adults ≥65 will likely need substance use disorder treatment in the coming years, given significant increases in both past-year cannabis use and cannabis use disorders found in multiple general population studies and across multiple demographic subgroups (e.g., Blacks and Whites) (Wu and Blazer 2011). A study using projections of NSDUH data similarly predicted that rates of substance use disorder will double from 2.8 million in 2002–2006 to 5.7 in 2020 among adults 50 and older in the USA because of the baby-boom cohort’s large size and high rates of substance use (Han et al. 2009).

### Limitations and future steps

These results come with some limitations, including that Washington’s population may have unique characteristics affecting generalizability, e.g., the prevalence of cannabis use in Washington is among the highest in the USA (Center for Behavioral Health Statistics and Quality 2018). The Washington Behavioral Risk Factor Surveillance System (BRFSS) found that past 30-day cannabis use increased from 5.8% in 2009 to 13.2% in 2016, which is similar to the changes we observed in past 12-month use (Everson et al. 2019). However, we are unable to compare our results to the BRFSS due to the different timeframes (past 30 days vs. past 12 months) and because this BRFSS analysis did not examine cannabis use among older individuals specifically.

Priority clinical subgroups, e.g., individuals with cannabis use disorders, may be under-represented because of their low prevalence in general population surveys. Outcomes are self-reported and may be influenced by reporting biases, such as acceptability of cannabis use or changes in the distribution of non-response. Other drug use, including prescription medications, was only asked in wave 6, precluding analyses of interactions between cannabis and other drug use due to small sample size. Related to sample size, some confidence intervals reported here are wide, reflecting low precision. Similarly, while the current results were not robust to all Benjamini-Hochberg and Bonferroni corrections, these particular *P*-value adjustment approaches are conservative, and our sample size is small and not powered to detect multiple significant associations under such strict conditions. Importantly, the significant differences in cannabis use across ages and recent increases in cannabis use among older adults observed in the current sample are similar to those published from other samples (Everson et al. 2019) and locations (Han and Palamar 2020), which reflects consistency and supports validity. Future studies should aim to reach more older individuals who use cannabis, e.g., through targeted recruiting at cannabis retail outlets.

The current study did not collect data regarding motivations for use or the CBD or THC content of products used. Selecting the ratio of CBD to THC can depend on the condition or motivation for use, e.g., chronic pain, and therefore may also vary according to age. Given that the use of cannabis to treat chronic pain conditions (Nugent et al. 2017) is increasing combined with the high prevalence of chronic pain conditions among older adults (Reid et al. 2015), motivations for use may include chronic pain. Ongoing studies are collecting more detailed data on motivations for cannabis use as well as CBD and THC preferences. Related, the current survey asked whether participants had a medical recommendation from a doctor for cannabis, but did not ask whether participants used cannabis for medical vs. recreational purposes. Given that all data were collected post-legalization, meaning participants could legally obtain cannabis without a medical recommendation, and given that individuals could use cannabis for medical purposes even without a medical recommendation, we are not able to accurately distinguish medical vs. recreational use with the current survey.

## Conclusions

The prevalence of cannabis use among older adults is rising. Our findings extend past results by examining cannabis use frequency, route of administration, and co-use with alcohol in this population. Among older adults living in Washington, a state with legal adult use cannabis, the prevalence of cannabis use increased among those aged 50–64 between 2014 and 2016, the prevalence of daily use is substantial, and oral administration and vaping are increasing. Identifying older adults who use cannabis can help inform public health and clinical efforts, e.g., through patient education regarding proper dosing and risks related to co-use with alcohol.

## Data Availability

Data are not publicly available per Institutional Review Board protocol.

## Abbreviations

US: United States
NSDUH: National Survey on Drug Use and Health
RRR: Relative risk ratio
BRFSS: Behavioral Risk Factor Surveillance System
